# Digital Coaching Using Smart Inhaler Technology to Improve Asthma Management in Patients With Asthma in Italy: Community-Based Study

**DOI:** 10.2196/25879

**Published:** 2022-11-02

**Authors:** Gabriele Rumi, G Walter Canonica, Juliet M Foster, Niels H Chavannes, Giuseppe Valenti, Rosario Contiguglia, Eleni Rapsomaniki, Janwillem W H Kocks, Dario De Brasi, Fulvio Braido

**Affiliations:** 1 Dipartimento di Scienze Mediche e Chirurgiche Fondazione Policlinico Universitario A Gemelli IRCCS Università Cattolica del Sacro Cuore – Medicina Interna Rome Italy; 2 Personalized Medicine Asthma & Allergy Clinic-Humanitas University & Research Hospital IRCCS Milan Italy; 3 Woolcock Institute of Medical Research University of Sydney Sydney Australia; 4 Department of Public Health and Primary Care Leiden University Medical Center Leiden Netherlands; 5 PTA Biondo ASP Palermo Palermo Italy; 6 Personalized Medicine Clinic of Respiratory Diseases Messina Italy; 7 BioPharmaceuticals Medical AstraZeneca Cambridge United Kingdom; 8 General Practitioners Research Institute Groningen Netherlands; 9 ASL Napoli 2 Nord Naples Italy; 10 Department of Internal Medicine University of Genova Genova Italy

**Keywords:** asthma control, asthma management, connected devices, digital health, eHealth, inhalers, maintenance and reliever therapy, mobile phone

## Abstract

**Background:**

Reliance on short-acting *β*-2 agonists and nonadherence to maintenance medication are associated with poor clinical outcomes in asthma. Digital health solutions could support optimal medication use and therefore disease control in patients with asthma; however, their use in community settings has not been determined.

**Objective:**

The primary objective of this study is to investigate community implementation of the Turbu+ program designed to support asthma self-management, including adherence to budesonide and formoterol (Symbicort) Turbuhaler, a combination inhaler for both maintenance therapy or maintenance and reliever therapy. The secondary objective is to provide health care professionals with insights into how patients were using their medication in real life.

**Methods:**

Patients with physician-diagnosed asthma were prescribed budesonide and formoterol as maintenance therapy, at a dose of either 1 inhalation twice daily (1-BID) or 2 inhalations twice daily (2-BID), or as maintenance and reliever therapy (1-BID and reliever or 2-BID and reliever in a single inhaler), and they received training on Turbu+ in secondary care centers across Italy. An electronic device attached to the patients’ inhaler for ≥90 days (data cutoff) securely uploaded medication use data to a smartphone app and provided reminders, visualized medication use, and motivational nudge messages. Average medication adherence was defined as the proportion of daily maintenance inhalations taken as prescribed (number of recorded maintenance actuations per day or maintenance inhalations prescribed per day) averaged over the monitoring period. The proportion of adherent days was defined as the proportion of days when all prescribed maintenance inhalations were taken on a given day. The Wilcoxon test was used to compare the proportion of adherent days between patients in the maintenance regimen and patients in the maintenance and reliever regimen of a given dose.

**Results:**

In 661 patients, the mean (SD) number of days monitored was 217.2 (SD 109.0) days. The average medication adherence (maintenance doses taken/doses prescribed) was 70.2% (108,040/153,820) overall and was similar across the groups (1-BID: 6332/9520, 66.5%; 1‑BID and reliever: 43,578/61,360, 71.0%; 2-BID: 10,088/14,960, 67.4%; 2-BID and reliever: 48,042/67,980, 70.7%). The proportion of adherent days (prescribed maintenance doses/doses taken in a given day) was 56.6% (31,812/56,175) overall and was higher with maintenance and reliever therapy (1-BID and reliever vs 1-BID: 18,413/30,680, 60.0% vs 2510/4760, 52.7%; *P*<.001; 2-BID and reliever vs 2-BID: 8995/16,995, 52.9% vs 1894/3740, 50.6%; *P*=.02). Rates of discontinuation from the Turbu+ program were significantly lower with maintenance and reliever therapy compared with maintenance therapy alone (*P*=.01).

**Conclusions:**

Overall, the high medication adherence observed during the study might be attributed to the electronic monitoring and feedback mechanism provided by the Turbu+ program.

## Introduction

Asthma control is suboptimal in approximately half of adult patients worldwide and is reflected by poorly controlled symptoms, impairment in daily activities, and increased use of health care resources [1-3]. Suboptimal asthma management can result from reliance on the use of short-acting *β*-2 agonists (SABAs) and poor adherence to prescribed medication, either intentionally (intelligent nonadherence) or unintentionally (erratic or unwitting nonadherence) [4-6]. Patients may also exhibit inconsistent patterns of adherence based on the presence or absence of symptoms (eg, underuse or total nonuse when asymptomatic) or on the types of medications prescribed (eg, overuse of SABA-reliever therapy and underuse of maintenance therapy) [5].

Overall, adherence to asthma medication is generally poor, with reported rates of nonadherence ranging from 30% to 70% [7], with adherence rates in community settings as low as 20%-30% [8-10]. Moreover, poor asthma control, including nonadherence to therapy, is associated with an increased risk of severe exacerbations, a reduction in health-related quality of life, and an increase in health care costs [2,11,12]. Consequently, there is a need for a more personalized and integrated approach to address this issue. Over the past decade, new technologies and innovations in digital health care have been introduced to support optimal medication use and disease management in patients with asthma. Notably, randomized controlled trials using these technologies have demonstrated the effectiveness of electronic medication use monitoring, including electronic reminders and feedback, in improving adherence and achieving asthma control [13-15]. However, the implementation of such technologies in community settings, where patients are not supported by the structure and commitment of a clinical trial, is yet to be determined.

**Figure 1:**
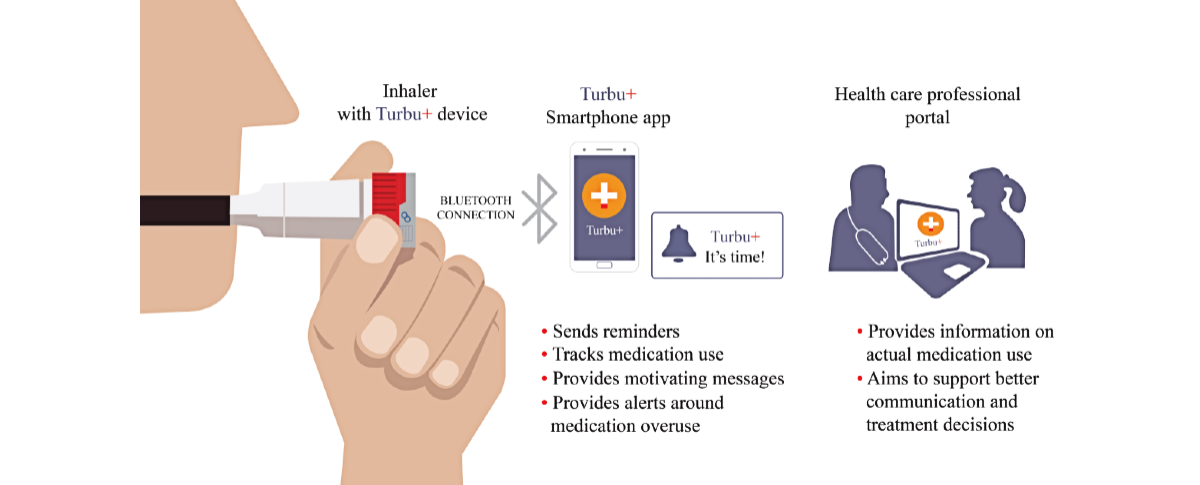
Digital technology used in the Turbu+ program (V2.1). Turbu+ device is manufactured by Adherium (NZ) Ltd.

One such innovative digital health technology is the Turbu+ program (Figure 1), which has been designed to improve adherence to prescribed therapy and increase asthma control, thereby supporting asthma management with budesonide and formoterol (Symbicort) Turbuhaler, a combination inhaler for both maintenance therapy or maintenance and reliever therapy (also known as maintenance and anti-inflammatory reliever therapy) in the community setting. The aim of the Turbu+ program is to assist patients in developing beneficial asthma self-management behaviors, either with their existing treatment or early in the use of a new treatment before less optimal patterns of inhaler use are established [16]. This can be achieved through digital coaching, with the provision of medication reminders [17,18], visualized medication use, and motivational messages [17] via Smartinhaler technology, which has been shown to improve adherence when compared with standard care as a part of asthma management [15]. Other research on attribute-framing effects reported more favorable responses to positive attribute frames than negative attribute frames, with positive frames evoking favorable associations in memory [19,20]. For example, results from a recent clustered, controlled pilot study of the Turbu+ program, which enrolled 80 patients in the Netherlands, reported that patients using Turbu+ were more likely to be adherent (4.5 times more likely to have a refill adherence of >80% prescribed; 95% CI 1.56-13.1) than those not using Turbu+. In patients with not well controlled asthma at baseline (Control of Allergic Rhinitis and Asthma Test scores <23 points), the odds of improvement after 6 months were 2.87 (95% CI 0.61-13.6) for the Turbu+ group compared with the control group [21]. In addition to supporting patients’ asthma self-management, the Turbu+ program also provides health care professionals with objective information on when and how often patients use their medication, which reinforces mutual sharing of information and treatment decision making.

Given the central role that treatment adherence plays in effective asthma control and the increasing importance of digital interventions in promoting engagement and self-management among users, we implemented the Turbu+ program in adult patients with asthma in Italy, where asthma is reported to affect approximately 2.5 million patients [22] and the rates of adherence to asthma therapy are low [23]. We hypothesized that the integration of asthma care into the everyday use of patients’ own mobile phones would enhance engagement with self-management, thereby improving adherence and overall asthma control.

## Methods

### Study Design and Patient Population

This was a community-based study that used electronic monitoring (Turbu+ device) and feedback via the Turbu+ app. Patients with physician-diagnosed asthma who were prescribed or receiving treatment with budesonide and formoterol maintenance therapy at a dose of either 1 inhalation twice daily (1-BID) or 2 inhalations twice daily (2-BID) or budesonide and formoterol maintenance and reliever therapy (1-BID and reliever or 2-BID and reliever in a single inhaler, also referred to as Symbicort maintenance and anti-inflammatory reliever therapy) were enrolled in the Turbu+ program by pulmonologists and allergists in public and private secondary care centers across Italy. Practices were representative of Italy and distributed across the country. To minimize interference with usual care, there was no formal protocol for the Turbu+ program; therefore, each physician used a personalized approach. However, based on individual conversations between physicians and patients, patients were enrolled for a range of different reasons: (1) to monitor the effectiveness of the treatment; (2) to help transition patients, either gradually or immediately, from an inhaled corticosteroid (ICS) and long-acting *β* agonist (LABA) treatment and a SABA to budesonide and formoterol inhaler maintenance and reliever therapy; (3) to monitor the appropriate use of budesonide and formoterol as reliever therapy, instead of SABA, within the context of maintenance and reliever therapy; and (4) to improve asthma control by enabling physicians to use real-life Turbu+ data in their dialog with their patients. Physicians registered patients into the Turbu+ program, which triggered the generation of a link that was sent to the patients’ email account. Patients could then download the app and log in.

In line with the noninterventional nature of this study design, minimal patient demographic information was collected (ie, age, sex, and treatment regimen). The data reported here are based on a statistical analysis of the aggregated and anonymized data captured by the Turbu+ program. The data underlying the findings described in this manuscript may be obtained in accordance with AstraZeneca’s data sharing policy [24].

### Inhaler Monitoring

Patients were provided with a Turbu+ device that could be attached to their Turbuhaler by their physicians. All patients received instructions on the Turbu+ program and the Turbu+ device. The device recorded the date and time of each inhalation and securely transferred use data to a companion smartphone app available to the patient and to a secure web-based portal accessible by the prescribing health care professional (Figure 1). Data management and storage were in line with General Data Protection Regulation (GDPR).

### Inhaler Reminders, Visualized Medication Use, Motivational Messages, and Personalized Asthma Management Discussions

The smartphone app enabled patients to track their inhalations and provided reminders (both adjustable and customized for weekdays and weekends) and motivational nudge messages (VOICE messages) for missed inhalations and to encourage patients to adhere to their prescribed treatment (eg, “What, no symptoms? Use your inhaler to help stay that way,” “Laugh louder, dance harder, run faster, help make asthma smaller,” and “Your inhaler was developed with you in mind. Use it daily to help leave asthma symptoms behind.”). The VOICE messages are short messages developed by Professor Rob Horne, University College London, by applying principles based on research into patient perceptions of treatment and drivers and barriers of treatment engagement [25]. In addition, the smartphone app provided tailored digital coaching that included updates on weekly medication use, alerts regarding treatment overuse, and changes to drug regimen or device utility messages (eg, to turn Bluetooth on and the battery status of the Turbu+ device).

Changes in treatment regimens were uncommon and handled by the patients’ physicians as per usual care. Only physicians were authorized to revise or update the treatment regimen in the smartphone app. However, the patients received a notification about the regimen change in the app. Data on the secure portal were available to physicians to support discussions on patterns of medication use between health care professionals and patients. Patients on multiple regimens or who changed treatment regimens during the study were excluded from the analysis.

### Analysis Population and Adherence Calculations

Patients who had joined the Turbu+ program before November 11, 2018, and had been enrolled in the program for at least 90 days were included in the analysis. The analysis was restricted to a minimum of 90 days to provide a sufficient number of patients with an adequate follow-up period. Daily maintenance inhalations were 2 inhalations per day for the 1-BID and 1-BID and reliever regimens and 4 inhalations per day for the 2-BID and 2-BID and reliever regimens. For the purpose of analysis, the scheduled maintenance doses were calculated as the first 2 inhalations of budesonide and formoterol Turbuhaler taken (actuation recorded) each day (day was defined as the time from 12 AM to 11:59 PM) by patients prescribed 1-BID and reliever regimens and the first 4 inhalations of budesonide and formoterol Turbuhaler taken by those prescribed 2-BID and reliever regimens. As it was not possible to entirely distinguish between inhalations taken for maintenance and inhalations taken for reliever therapy, certain assumptions were made for the purpose of analysis. On the basis of the total number of inhalations per day, if the maintenance dose was exceeded, it was considered as a reliever dose. Average medication adherence, the proportion of adherent days, and full adherence and zero adherence days were all analyzed (definitions are provided in Table 1).

Adherence by morning or evening was not analyzed, but the average morning and evening medication use times were plotted. The day of the first inhalation was considered as day 1 for the analysis. Consequently, patients with a medication delay after commencement of use of the smartphone app (defined as the gap between the start of using the app and the start of the first medication) were not excluded from the analysis.

The number of high-use days (>12 inhalations per day) was analyzed. On the basis that adherence in the first few weeks of the Turbu+ program was expected to be higher compared with long-term use, adherence in the first 15 days (proportion of maintenance inhalations taken over this period) was used to categorize patients into low (0% to <70%), medium (70% to <90%), and high (≥90%) adherence groups. These cutoffs were chosen to obtain an approximately equal number of patients in each group (<70% was considered as low adherence). Kaplan-Meier curves were then computed starting from >15 days.

Kaplan-Meier curves were used to analyze discontinuation from the Turbu+ program over time, with discontinuation from the program defined as the last recorded treatment for a patient more than 30 days before the date of data cutoff; the discontinuation date was the date of the last dose. All other patients (ie, patients who continued to receive treatment during the last 30 days before data cutoff) were assumed to be continuing the treatment, and these patients were censored on the date of the last recorded dose intake. Discontinuation from the Turbu+ program merely meant that no data were received in the app, with patients potentially having the app closed; it did not automatically mean discontinuation of the medication inhaler.

**Table 1:** Adherence definitions used in the study.

Adherence	Definition
Average medication adherence	Proportion of daily maintenance inhalations taken as prescribed (number of recorded maintenance actuations per day/number of maintenance inhalations prescribed per day^a,b^) and averaged over the monitoring period
Fully adherent days	Proportion of days during which all prescribed maintenance inhalations were taken (ie, recorded as actuated) in a given day
Zero adherence day	No inhalations taken in a given day (ie, a zero-dose day)

^a^For the purpose of analysis, the scheduled maintenance inhalations were calculated as the first 2 inhalations of budesonide and formoterol Turbuhaler taken each day by patients prescribed 1 inhalation twice daily and a reliever and the first 4 inhalations taken by those prescribed 2 inhalations twice daily and a reliever.

^b^Day was defined as the time from midnight to 11:59 PM.

### Statistical Analyses

Mean adherence was compared by using an analysis of variance model; adherence plots were based on moving averages, and the trend curves were smoothed using 3- or 5-day moving averages. The Wilcoxon test was used to compare the proportion of adherent days between patients in the maintenance regimen and patients in the maintenance and reliever regimen. A Cox model was designed to evaluate factors that most contributed to discontinuation of using the app. A *P* value of <.05, computed using the log-rank test, indicated that the differences among the Kaplan-Meier curves were statistically significant when compared using an analysis of variance model.

## Results

### Patient Demographics

Of the 1575 patients enrolled in the Turbu+ program, 661 patients who had been followed up in the program for ≥90 days (1-BID, n=56; 1-BID and reliever, n=361; 2-BID, n=44; and 2-BID and reliever, n=200) were included in the analysis (Table 2). Patients were aged between >18 years and <75 years, with the largest proportion of patients (165/661, 25.1%) aged between 46 years and 55 years. There was a similar proportion of female and male patients (female: 356/658, 54.1%; ranging from 45.5% to 64.3% across the 4 treatment groups).

Overall, the mean (SD) number of days monitored was 217.2 (SD 109.0) days: 1-BID: 235.7 (SD 126.0) days, 1-BID and reliever: 215.4 (SD 106.8) days, 2-BID: 181.9 (SD 104.4) days, and 2-BID and reliever: 223.0 (SD 107.8) days. The median (IQR) number of inhalations taken per day were as follows: 1-BID: 2 (IQR 1-2) inhalations, 1‑BID and reliever: 2 (IQR 1-2) inhalations, 2-BID: 4 (IQR 2-4) inhalations, and 2-BID and reliever: 4 (IQR 2-4) inhalations.

**Table 2:** Demographic characteristics of patients who remained in the program for ≥90 days and who discontinued earlier. Patients with missing age or sex details were excluded.

Characteristic				<90 days^a^		≥90 days		*P* value^b^	
Number of patients, n (%)				914 (58)		661 (42)		N/A^c^	
**Regimen, n (%)**									<.001
	1-BID^d^		137 (15)		56 (8.5)				
	1-BID and reliever		491 (53.7)		361 (54.6)				
	2-BID^e^		56 (6.1)		44 (6.7)				
	2-BID and reliever		230 (25.2)		200 (30.3)				
**Sex, n (%)**									.66
	Male		408 (44.6)		302^f^ (45.9)				
	Female		506 (55.4)		356^f^ (53.9)				
**Age (years), n (%)**									<.001
	<18		90 (9.9)		44 (6.7)				
	18-25		186 (20.6)		91 (13.8)				
	26-35		170 (18.8)		111 (16.9)				
	36-45		151 (16.7)		117 (17.8)				
	46-55		153 (16.9)		165 (25.1)				
	56-65		96 (10.6)		86 (13.1)				
	66-75		50 (5.5)		36 (5.5)				
	>75		9 (1)		8 (1.2)				
**Age group (years), n (%)**									<.001
	<36		446 (48.8)		246 (37.2)				
	36-55		304 (33.3)		282 (42.7)				
	>55		164 (17.9)		133 (20.1)				
**Age group per sex (years), n (%)**									<.001
	**Female**								
		<36	216 (23.6)		144^f^ (21.9)				
		36-55	188 (20.6)		149^f^ (22.6)				
		>55	102 (11.2)		63^f^ (9.6)				
	**Male**								
		<36	230 (25.2)		102^f^ (15.5)				
		36-55	116 (12.7)		133^f^ (20.2)				
		>55	62 (6.8)		67^f^ (10.2)				

^a^<90 days: patients who discontinued <90 days after the first dose.

^b^*P* values were based on analysis of variance tests for continuous variables and the chi-square test for categorical variables.

^c^N/A: not applicable.

^d^1-BID: 1 inhalation twice daily.

^e^2-BID: 2 inhalations twice daily.

^f^N=658.

### Average Medication Adherence

**Figure 2:**
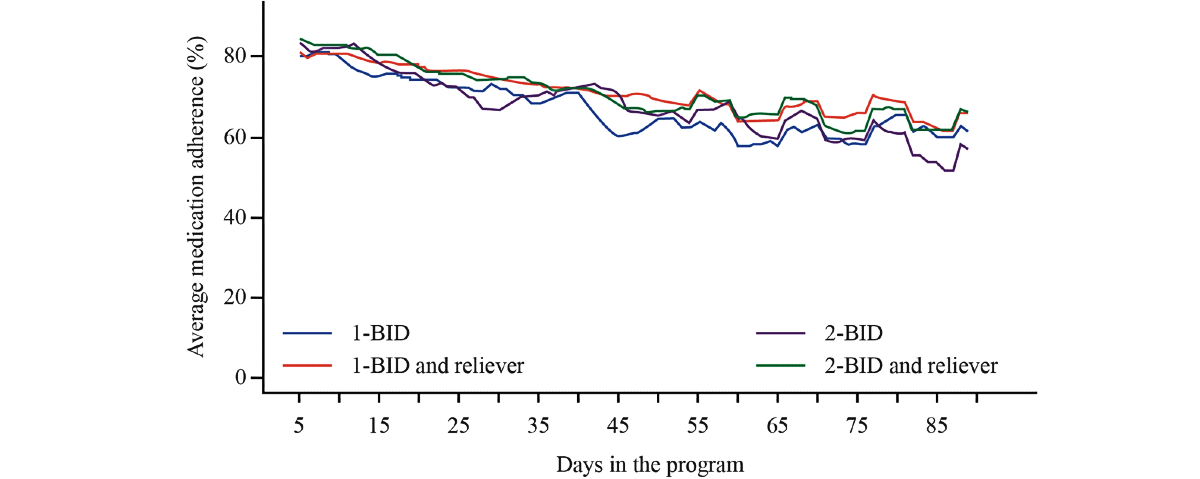
Average medication adherence by regimen and number of days in the program. Graphs are based on 5-day moving averages. Overall, the average medication adherence was 70.2% (1 inhalation twice daily [1-BID]: 66.5%, 1-BID and reliever: 71.0%, 2 inhalations twice daily [2-BID]: 67.4%, and 2-BID and reliever: 70.7%). 1-BID: 1 inhalation twice daily; 2-BID: 2 inhalations twice daily.

Average medication adherence to maintenance treatment (maintenance doses taken/doses prescribed) showed similar trends across the 4 treatment groups over time (Figure 2; Multimedia Appendix 1). Overall, average medication adherence was 70.2% (108,040/153,820): 1-BID: 66.5% (6332/9520), 1‑BID and reliever: 71.0% (43,578/61,360), 2-BID: 67.4% (10,088/14,960), and 2-BID and reliever: 70.7% (48,042/67,980).

**Figure 3:**
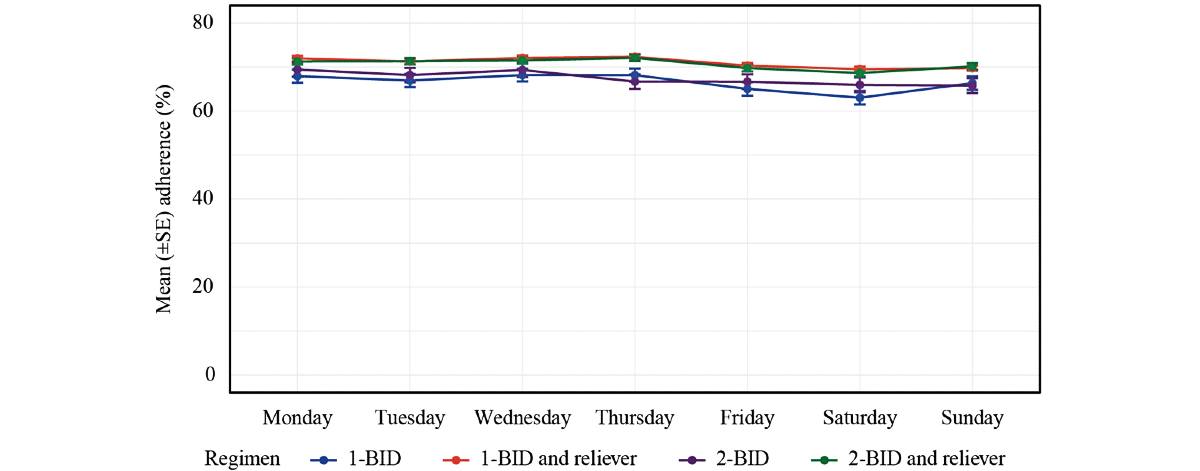
Adherence by weekday based on regimen. Data correspond to the mean per regimen and weekday during the first 90 days. 1-BID: 1 inhalation twice daily; 2-BID: 2 inhalations twice daily.

In exploratory analyses, the mean medication adherence was comparable for female and male patients (female, n=356, 70.8%; male, n=302, 69.9%; *P*=.65), with the lowest adherence observed in the youngest age group (Table 3). In addition, compared with other weekdays, adherence was lower on Fridays and weekends among all 4 treatment groups (Figure 3).

**Table 3:** Mean adherence by age and sex.

Characteristic				Patients, n		Percentage of adherence^a^, mean (SD)		*P* value^b^		
**Sex**										.65
	Female		356		70.7 (23.1)					
	Male		302		69.8 (25.7)					
**Age (years)**										.02
	<18		44		60.3 (25.7)					
	18-25		91		66.3 (24.3)					
	26-35		111		69.7 (22.6)					
	36-45		117		71.1 (24.5)					
	46-55		165		73 (22.5)					
	56-65		86		75.6 (25)					
	66-75		36		67 (29.6)					
**Age group (years)**										.02
	<36		246		66.8 (23.9)					
	36-55		282		72.2 (23.3)					
	>55		133		72.8 (26.4)					
**Age group per sex (years)**										.05
	**<36**									
		Female	144		66 (24.3)					
		Male	102		67.8 (23.4)					
	**36-55**									
		Female	149		72.9 (21.8)					
		Male	133		71.4 (25)					
	**>55**									
		Female	63		76 (21.7)					
		Male	67		69.8 (30)					

^a^Adherence was restricted to patients with ≥90 days since the start of medication; data includes day 90.

^b^*P* values are based on analysis of variance tests for continuous variables and the chi-square test for categorical variables.

### Proportion of Patients With Fully Adherent Days

**Figure 4:**
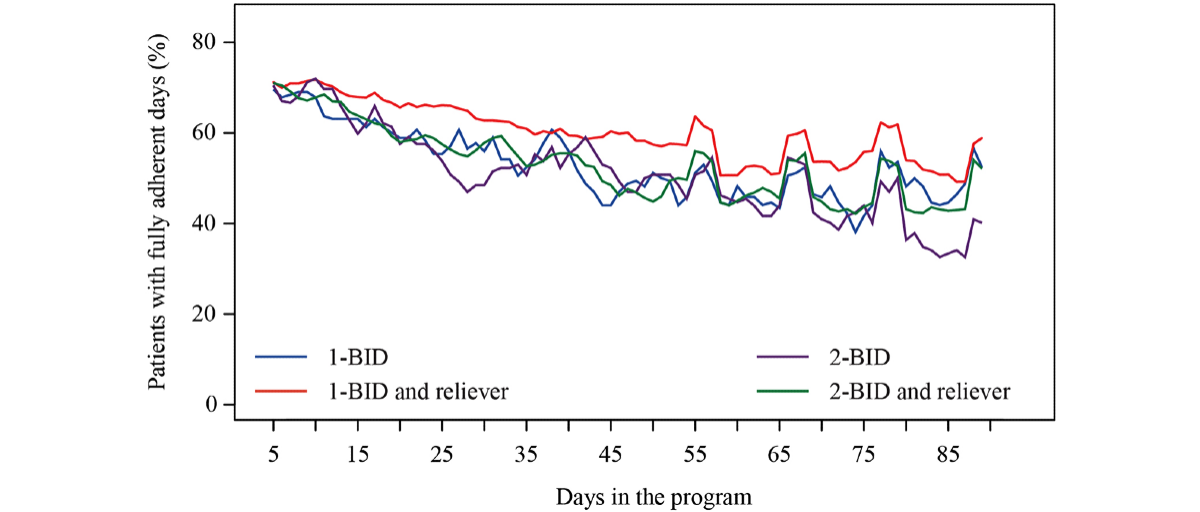
Proportion of patients with fully adherent days, by regimen and number of days in the program. Graphs are based on 3-day moving averages. The mean number of fully adherent days were as follows: 1 inhalation twice daily (1-BID): 60.0%, 1-BID and reliever: 52.7%, 2 inhalations twice daily (2-BID): 52.9%, and 2-BID and reliever: 50.6%. 1-BID: 1 inhalation twice daily; 2-BID: 2 inhalations twice daily.

The proportion of patients with fully adherent days (days during which all prescribed maintenance inhalations were taken) varied across the 4 treatment groups (Figure 4; Multimedia Appendix 2). The proportion of patients with fully adherent days was 56.6% (31,812/56,175) overall and was higher with maintenance and reliever therapy than with budesonide/formoterol maintenance therapy (1-BID and reliever vs 1-BID: 18,413/30,680, 60.0% vs 2510/4760, 52.7%, respectively; *P*<.001 and 2-BID and reliever vs 2‑BID: 8995/16,995, 52.9% vs 1894/3740, 50.6%, respectively; *P*=.02).

### Proportion of Zero Adherence Days

Overall, the proportion of zero adherence days (no inhalations per day) was 17.0% (9534/56,175) and was similar between the treatment regimens: 1-BID and reliever versus 1-BID: 18.0% (5515/30,680) versus 19.7% (938/4760), respectively (*P*=.07); and 2‑BID and reliever versus 2-BID: 14.5% (2462/16,996) versus 16.6% (619/3740), respectively (*P*=.21; Multimedia Appendix 3).

### Inhalations Taken at Specific Times of the Day

**Figure 5:**
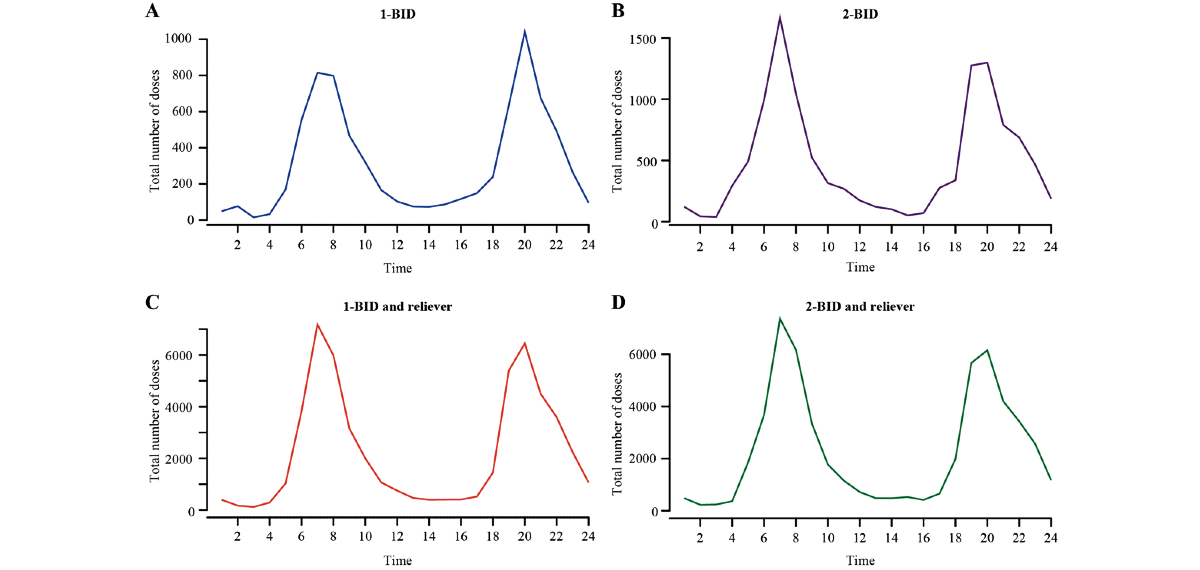
Inhalations taken at specific time of the day (24-hour clock) during the first 90 days. The y-axis includes the total number of inhalations (ie, the sum of the number of maintenance and reliever inhalations taken) per given time point across all days in the 90-day period and across all patients. 1-BID: 1 inhalation twice daily; 2-BID: 2 inhalations twice daily.

For each regimen, the time of morning inhalation was generally between 6 AM and 10 AM and evening inhalation was between 6 PM and 10 PM (Figure 5).

### High-Use Days

Of the 143,562 person-days in the program, the proportion of high-use days (>12 inhalations per day) was low (approximately 0.03/100 person-days). Overall, 24 patients recorded >12 inhalations per day on 42 days (mean 1.75 days; median 1 day, range 1-10 days; Multimedia Appendix 4; Table 4). Many high-use days occurred in the first week (17 of the 42 high-use days occurred between days 1 and 4).

**Table 4:** High-use days: >12 inhalations a day (any day).

Regimen	Patients (N=661)	Days	Person-days
	n (%)	n (%)	n
1-BID^a^	1 (0.15)	3 (0.02)	13,198
1-BID and reliever	10 (1.51)	12 (0.02)	77,764
2-BID^b^	3 (0.45)	3 (0.04)	8004
2-BID and reliever	10 (1.51)	24 (0.05)	44,596

^a^1-BID: 1 inhalation twice daily.

^b^2-BID: 2 inhalations twice daily.

### Discontinuation From the Turbu+ Program

**Figure 6:**
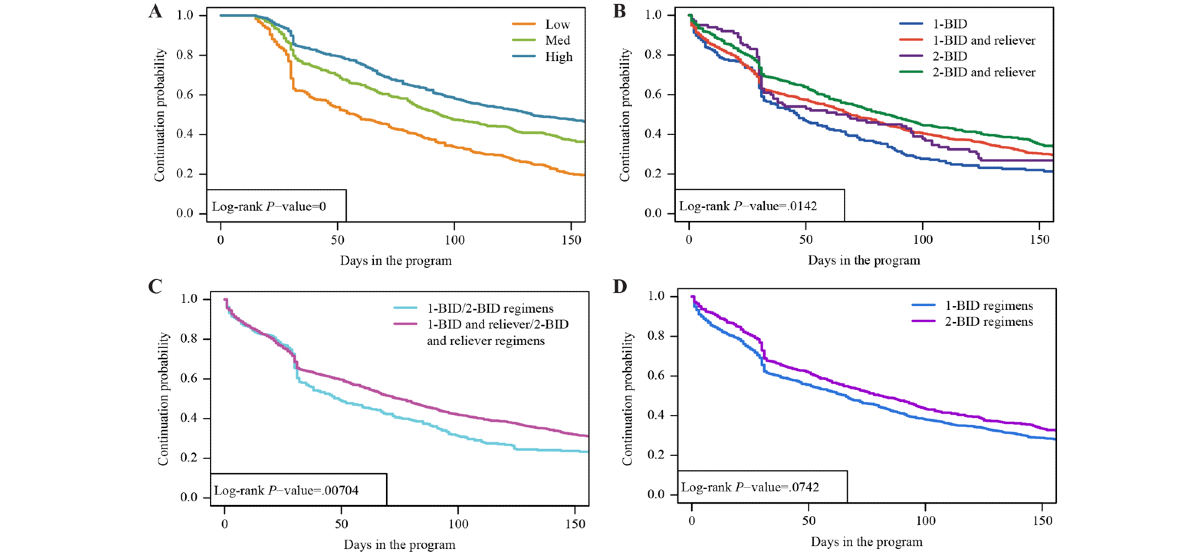
Time to discontinuation from the Turbu+ program, (A) by adherence group in the first 15 days (low, medium, or high) and (B, C, and D) by regimen. Low: n=385; medium: n=419; high: n=512 (patients with <15 days of follow-up were excluded from this analysis). 1-BID: 1 inhalation twice daily; 2-BID: 2 inhalations twice daily.

Of all the patients enrolled in the Turbu+ program, patients who were categorized in the low adherence group in the first 15 days of the program discontinued the Turbu+ program at much faster rates up to day 30 compared with patients categorized in the medium adherence and high adherence groups. A sharp increase in discontinuation from the Turbu+ program was observed between days 30 and 40. After day 30, the discontinuation rates were similar across the 3 adherence groups (Figure 6). Overall, the rates of discontinuation from the program were significantly higher with maintenance therapy alone than with maintenance and reliever therapy (Figure 6B and 6C). Discontinuation rates were similar in the 1-BID and 2-BID regimens (Figure 6D). For those patients receiving maintenance and reliever therapy, similar proportions within each treatment group continued in the program after 90 days compared with patients who discontinued earlier than 90 days (1-BID and reliever: 361/661, 54.6% vs 491/914, 53.7%; 2-BID and reliever: 200/661, 30.3% vs 230/914, 25.2%; Table 2).

Treatment regimen and sex did not affect the overall discontinuation from the Turbu+ program. However, patient age and adherence in the first 15 days had a significant effect. Patients in the <36 years age group were more likely to discontinue the program than those in the ≥36 years age group (36-55 years: hazard ratio [HR] 0.75; 95% CI 0.66-0.86; *P*<.001 and >55 years: HR 0.76; 95% CI 0.64-0.90; *P*=.001). Likewise, patients categorized in the low adherence group were more likely to discontinue the program than those categorized in the medium adherence (HR 0.63; 95% CI 0.54-0.73; *P*<.05) or high adherence (HR 0.50; 95% CI 0.43-0.58; *P*<.05) groups (Multimedia Appendices 5 and 6).

## Discussion

### Principal Findings

The Turbu+ program, designed to support asthma self-management and adherence to budesonide and formoterol Turbuhaler in a community setting, reported an average medication rate of 70%. This program conforms to the high-quality real-life respiratory research recommendations of the Respiratory Effectiveness Group [26]. There was no formal protocol for enrollment in the Turbu+ program; instead, physicians used a personalized approach to recruitment based on individual conversations between physicians and patients. Patients were enrolled for a multitude of reasons, taking patients’ individual needs and preferences into consideration. This enabled patients to develop the knowledge and skills required to effectively manage and make informed decisions about their asthma. There is a wealth of evidence highlighting that a patient’s ability to self-manage asthma improves clinical outcomes, patient satisfaction, and quality of life [27].

The implementation of the Turbu+ program in patients with asthma in Italy was continued for more than 90 days in over 600 patients across a wide range of age groups, suggesting that, for those who engaged in the program, it was well accepted and feasible for use by adolescents and young adults through to older adults. Moreover, to the best of our knowledge, this is the first study to provide detailed patterns of daily use of budesonide and formoterol maintenance therapy or budesonide and formoterol maintenance and reliever therapy in a real-life community setting.

The adherence rate observed in this study was considerably higher than that generally reported in Italy [22,23]. Results from the cross-sectional phase of the PRISMA (Prospective Study on Asthma Control) study, which investigated the level of asthma control in 2853 patients recruited from 56 respiratory clinics in Italy, indicated that although 64.4% of patients had controlled asthma, over one-third of patients were still uncontrolled or partly controlled. The main reason for poor asthma control, as indicated by the treating physicians, was low adherence to treatment in 43.3% of patients [23]. Similarly, the majority of 174 Italian allergists who completed a survey that was available on the website of Società Italiana di Allergologia, Asma Immunologia Clinica between April 2015 and October 2015 considered poor adherence to therapy to be an important cause of symptom worsening, with 37% believing that it was the prevalent cause [22]. The adherence rates observed in our study were generally higher than those reported in other studies, where low adherence rates to ICS and LABAs have generally been reported. Indeed, results from an observational study based on the Veneto region Drug Regulatory Agency database in Italy reported suboptimal adherence (defined as <80% prescriptions issued) in 57.6% of patients with asthma prescribed ICS and LABA in a 6-month period before the start of biologic treatment [28]. Using the same threshold of <80% prescriptions, 62.4% of patients prescribed combined ICS and LABA inhalers over the previous 12 months had suboptimal adherence in a retrospective observational study in the United Kingdom [29]. Results from a multinational, real-world observational study in Europe showed that only 34.4% of patients receiving ICS and LABA therapy reported high adherence (score=8) based on the Morisky Medication Adherence Scale questionnaire over a 3-month period [30]. However, results from another retrospective new-user active-comparator database study, using the IQVIA Medical Research Database in the United Kingdom, reported treatment persistence for ICS and LABA therapies ranging from 53% to 69% at 12 months, depending on the inhaler used [31]. Although direct comparisons of adherence rates with other studies could not be made because of variations in the measurement of adherence calculations, differences in study designs and duration, and the innovative community design of this study, our study results demonstrate that the use of the Turbu+ program, through the provision of reminders, visualized medication use, and motivational messages to patients as part of digital coaching, improves adherence (>70%) to inhaled medications. Because targeting adherence interventions to patients with the most to gain may improve asthma outcomes and optimize cost-effectiveness [32], further research is required to determine who will benefit most from the Turbu+ program. In addition, an evaluation of the cost-effectiveness of the Turbu+ program in the community setting may be required to ensure its widespread adoption. Recent work undertaken by the @IT-2020 project reported high rates of adherence using a blended care approach (face-to-face visits with internet-based support technologies) in patients from Italy with seasonal allergic rhinitis [33]. Incorporating such a blended approach with the Turbu+ program may further improve adherence.

Overall, a large proportion of patients (n=914) did not continue in the Turbu+ program for 90 days or more. The sharp increase in discontinuation from the Turbu+ program observed between days 30 and 40 was likely due to the fact that patients were transitioning to another inhaler, as most devices contain a month’s supply of medication. However, it is important to note that anecdotal evidence indicated that some physicians used the Turbu+ program only for a limited period to assess clinical effectiveness and appropriate use of budesonide and formoterol maintenance and reliever therapy, and once reassured about the appropriateness of treatment, they agreed that patients could discontinue the Turbu+ program. The long-term Turbu+ program used in this analysis may not appeal to all patients or its design may not be engaging enough to encourage enduring use. Additional features, such as symptom tracking to encourage asthma self-management, may have resulted in greater engagement with the program. However, data generated from the patients who discontinued from the Turbu+ program provide valuable information for future research to understand what could be done to increase user engagement to improve patient retention in the program.

Indeed, the results demonstrated that lower levels of adherence in the first 2 weeks and younger age (<36 years) were predictors of patient discontinuation from the Turbu+ program. This reinforces the need for clinicians to spend more time with their patients during the early weeks, especially with the younger patient population, to assess medication use and establish the right patient behavior early on in a new treatment [16]. Moreover, the rapid identification of patients with poor adherence enables early interventions by clinicians that could reduce the risks associated with long-term poor adherence, such as exacerbations and subsequent increased health care utilization [2,11,12], thereby improving asthma outcomes. The web-based portal provided physicians with objective, detailed, and valuable insights into their patients’ medication use versus the prescribed regimen, thereby ensuring that subsequent conversations were based on their actual medication use. Although the frequency with which clinicians viewed the medication use data was not assessed in this study, the ability to access such information via the secure web-based portal would be advantageous in this regard.

Although female patients have a greater number of physician visits and, thus, a higher likelihood of having an asthma action plan than male patients [34], studies exploring the relationship between sex and adherence to asthma treatment have provided conflicting results [35,36]. Similarly, this study failed to find any significant difference between the sexes, with mean medication adherence rates similar between male and female patients enrolled in the Turbu+ program. However, as expected, a decrease in adherence was generally observed on weekends, thereby confirming previous reports that weekends and holidays disrupt medication routines [5,37].

Interestingly, the proportion of fully adherent days was higher and the rates of discontinuation from the Turbu+ program were lower with maintenance and reliever therapy than with maintenance therapy alone. This may demonstrate a preference for a patient-centric regimen that enables patients to engage with their treatment and condition. This is in line with the findings of the findings of the PRACTICAL (PeRsonalised Asthma Combination Therapy: with Inhaled Corticosteroid And fast-onset Long-acting beta agonist) study, a randomized controlled trial that assessed patients’ preferences for symptom-driven maintenance plus reliever treatment or regular maintenance treatment in patients with mild to moderate asthma [38]. Overall, a significantly higher proportion of patients randomized to maintenance and reliever therapy preferred their therapy than those randomized to maintenance therapy alone. This finding further demonstrates patients’ preference for rapid symptom relief and flexible regimens over which they are in control, highlighting the importance of symptom-driven medication use in addition to medication adherence [38]. In this study, however, it was not possible to entirely distinguish between inhalations taken for maintenance and reliever therapy, as this information was not recorded by the patients.

The Turbu+ program helped patients follow a regular schedule of medication, as evidenced by the majority of fixed inhalations being taken between 6 AM and 10 AM and between 6 PM and 10 PM. Reassuringly, the Turbu+ program can provide information on the potential overuse of medication. Furthermore, only 24 patients recorded >12 inhalations per day with a mean of 1.75 day, indicating that the risk of overuse was minimal. High-use days were more common during the first week of use, possibly due to higher patient engagement on account of the novelty factor or potentially due to patient experimentation with the device to enhance understanding, which may have resulted in a higher number of actuations being registered in the app (it cannot be definitively concluded that patients actually took the medication). However, the number of high-use days gradually decreased and stabilized after the first 2 weeks.

### Limitations

The uniqueness of the design of this community-based Turbu+ program may have led to some of its limitations. Patients enrolled in the Turbu+ program for less than 90 days were excluded from the analysis to enable an examination of changes in patient behavior and its effect on adherence and asthma control after 90 days in the program. Reasons for discontinuation were not collected from those who discontinued from the Turbu+ program before 90 days; therefore, data for those patients who may have continued with their medication but discontinued from the Turbu+ program were also not captured. Data from the first 15 days in the program were excluded from the analysis to account for learning the program and technology. Limited baseline data were available on patient demographic and clinical characteristics because it was not part of the design of this physician-led program conducted in community-based clinical settings. Given that misdiagnosis of asthma is commonly reported, in part due to underuse of spirometry, it may have been beneficial to evaluate patients to accurately define their disease and to identify any symptoms caused by other factors before enrollment in the Turbu+ program [39-42]. Although the study population is suggestive of patients with moderate-to-severe asthma (Global Initiative for Asthma steps 3-5), formal asthma severity was not captured in the database. As a result of the study design, sizes of the treatment groups were not homogeneous; there were fewer patients receiving maintenance therapy alone compared with those receiving maintenance and reliever therapy. In addition, the strength of the doses was not captured. There was also a lack of randomization and the absence of a control or comparator group. Baseline adherence data at the start of using the Turbu+ program and reasons for adherence or nonadherence were not collected. Consequently, it is not known whether patient adherence was modified following enrollment in the Turbu+ program. However, these limitations are not unexpected, given the numerous challenges that have been previously observed in recruitment for studies using mobile phone technology, highlighting the need to pilot the recruitment process and design such trials accordingly [43]. However, with recent advances and the adoption of digital health technology during the COVID-19 pandemic, recruitment challenges in future studies may not be an issue.

### Conclusions

To the best of our knowledge, this is the first study to investigate the community implementation of the Turbu+ program and provide real-life information on the use of budesonide and formoterol Turbuhaler in over 600 patients who had been enrolled in the program for at least 90 days. The Turbu+ program provided patients with an effective tool to improve self-management of their asthma by tracking medication use and providing them with reminders and motivational messages. It provided health care professionals insights into their patients’ medication use, which supported treatment optimization and facilitated conversation between the health care professional and the patient. Additional prospective research on the Turbu+ program is required to understand who would benefit the most from this type of community-based adherence support and how to enhance user engagement. A longer follow-up period and an evaluation of asthma-related outcomes will further determine the contribution of the Turbu+ program in optimizing asthma outcomes.

## Appendix

Multimedia Appendix 1 Average medication adherence, by regimen and time. Graphs are based on 5-day moving averages. The dotted lines show the spread of data (±SE). 1-BID: 1 inhalation twice daily; 2-BID: 2 inhalations twice daily.

Multimedia Appendix 2 Proportion of patients with fully adherent days, by regimen and days in the program. Graphs are based on 3-day moving averages. The dotted lines show the spread of data (±SE). 1-BID: 1 inhalation twice daily; 2-BID: 2 inhalations twice daily.

Multimedia Appendix 3 Proportion of patients with zero adherence days, by regimen and days in the program. Graphs are based on 5-day moving averages. The dotted lines show the spread of data (±SE). 1-BID: 1 inhalation twice daily; 2-BID: 2 inhalations twice daily.

Multimedia Appendix 4 High-use days: a histogram of >12 inhalations a day (any day). This includes all patients, and one patient may be considered multiple times if he/she exceeded >12 inhalations for >1 day.

Multimedia Appendix 5 Cox model evaluating factors that contributed the most to discontinuation from the Turbu+ program. **P*<.01; ***P*<.001; 1-BID: 1 inhalation twice daily; 2-BID: 2 inhalations twice daily; High=High adherence (≥90%) at day 1 to day 15 in the program; Medium=medium adherence (70%-<90%) at day 1 to day 15 in the program.

Multimedia Appendix 6 Cox model evaluating factors that contributed the most to discontinuation from the Turbu+ program.

## Abbreviations

1-BID: 1 inhalation twice daily
2-BID: 2 inhalations twice daily
GDPR: General Data Protection Regulation
HR: hazard ratio
ICS: inhaled corticosteroid
LABA: long-acting *β* agonist
PRACTICAL: PeRsonalised Asthma Combination Therapy: with Inhaled Corticosteroid And fast-onset Long-acting beta agonist
PRISMA: Prospective Study on Asthma Control
SABA: short-acting *β*-2 agonist
