# Correction: Digital Coaching Using Smart Inhaler Technology to Improve Asthma Management in Patients With Asthma in Italy: Community-Based Study

**DOI:** 10.2196/44532

**Published:** 2022-11-25

**Authors:** Gabriele Rumi, G Walter Canonica, Juliet M Foster, Niels H Chavannes, Giuseppe Valenti, Rosario Contiguglia, Eleni Rapsomaniki, Janwillem W H Kocks, Dario De Brasi, Fulvio Braido

**Affiliations:** 1 Dipartimento di Scienze Mediche e Chirurgiche Fondazione Policlinico Universitario A Gemelli IRCCS Università Cattolica del Sacro Cuore – Medicina Interna Rome Italy; 2 Personalized Medicine Asthma & Allergy Clinic-Humanitas University & Research Hospital IRCCS Milan Italy; 3 Woolcock Institute of Medical Research University of Sydney Sydney Australia; 4 Department of Public Health and Primary Care Leiden University Medical Center Leiden Netherlands; 5 PTA Biondo ASP Palermo Palermo Italy; 6 Personalized Medicine Clinic of Respiratory Diseases Messina Italy; 7 BioPharmaceuticals Medical AstraZeneca Cambridge United Kingdom; 8 General Practitioners Research Institute Groningen Netherlands; 9 ASL Napoli 2 Nord Naples Italy; 10 Department of Internal Medicine University of Genova Genova Italy

In “Digital Coaching Using Smart Inhaler Technology to Improve Asthma Management in Patients With Asthma in Italy: Community-Based Study” (JMIR Mhealth Uhealth 2022;10(11):e25879), the authors noted one error.

In the PDF version of the originally published article, Clementina Columbro’s name was inadvertently included in the list of authors as the fifth author due to a technical error.

The list of authors of the paper and their respective affiliations have now been corrected in the PDF version of the article as follows:

Gabriele Rumi^1^, MD; G Walter Canonica^2^, MD; Juliet M Foster^3^, PhD; Niels H Chavannes^4^, PhD; Giuseppe Valenti^5^, MD; Rosario Contiguglia^6^, MD; Eleni Rapsomaniki^7^, PhD; Janwillem W H Kocks^8^, PhD; Dario De Brasi^9^, MD; Fulvio Braido^10^, MD

^1^Dipartimento di Scienze Mediche e Chirurgiche, Fondazione Policlinico Universitario A Gemelli IRCCS, Università Cattolica del Sacro Cuore – Medicina Interna, Rome, Italy

^2^Personalized Medicine Asthma & Allergy Clinic-Humanitas University & Research Hospital, IRCCS, Milan, Italy

^3^Woolcock Institute of Medical Research, University of Sydney, Sydney, Australia

^4^Department of Public Health and Primary Care, Leiden University Medical Center, Leiden, Netherlands

^5^PTA Biondo ASP Palermo, Palermo, Italy

^6^Personalized Medicine Clinic of Respiratory Diseases, Messina, Italy

^7^BioPharmaceuticals Medical, AstraZeneca, Cambridge, United Kingdom

^8^General Practitioners Research Institute, Groningen, Netherlands

^9^ASL Napoli 2 Nord, Naples, Italy

^10^Department of Internal Medicine, University of Genova, Genova, Italy

The correction will appear in the online version of the paper on the JMIR Publications website on November 25, 2022, together with the publication of this correction notice. Because this was made after submission to PubMed, PubMed Central, and other full-text repositories, the corrected article has also been resubmitted to those repositories.

