# Supplementary figures and images for: Digital Coaching Using Smart Inhaler Technology to Improve Asthma Management in Patients With Asthma in Italy: Community-Based Study
Source: JMIR Mhealth Uhealth. 2022 Nov 2;10(11):e25879. doi: 10.2196/25879 (PMC9669888; doi:10.2196/25879)

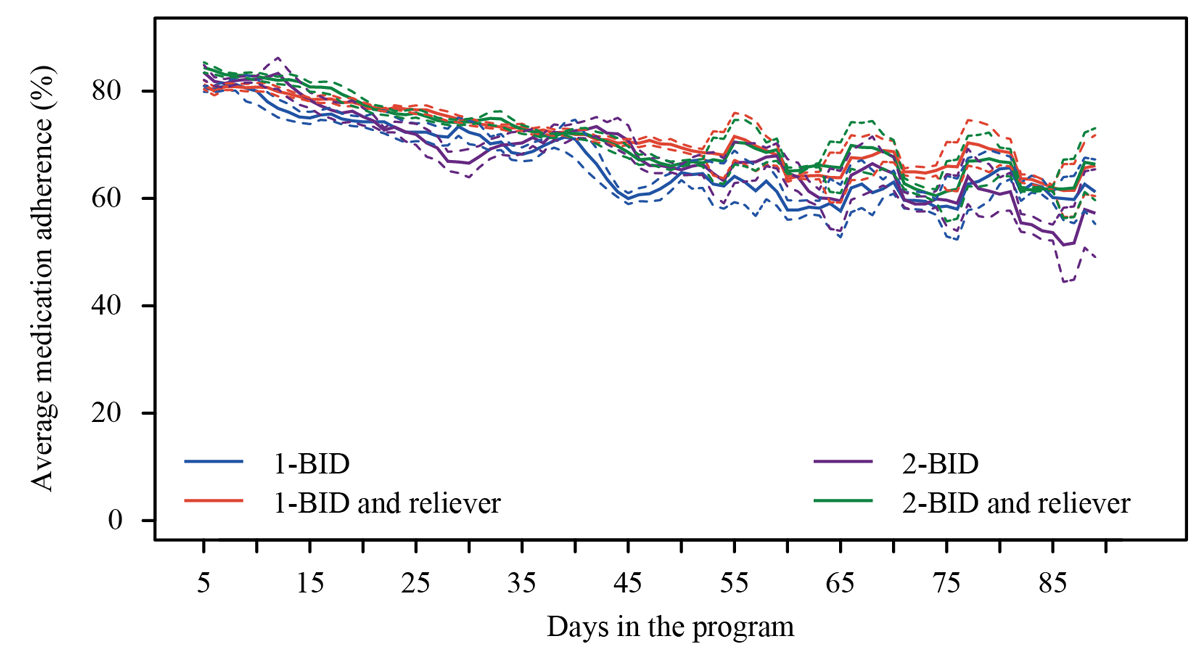

Supplement: Multimedia Appendix 1 [file mhealth_v10i11e25879_app1.png]

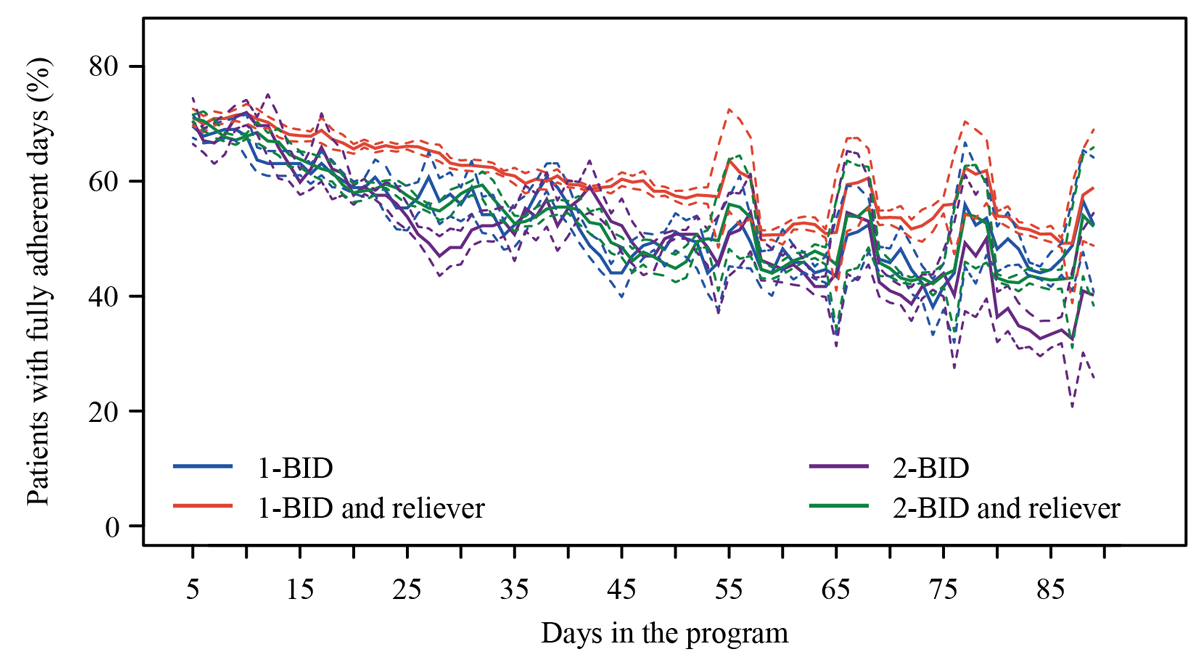

Supplement: Multimedia Appendix 2 [file mhealth_v10i11e25879_app2.png]

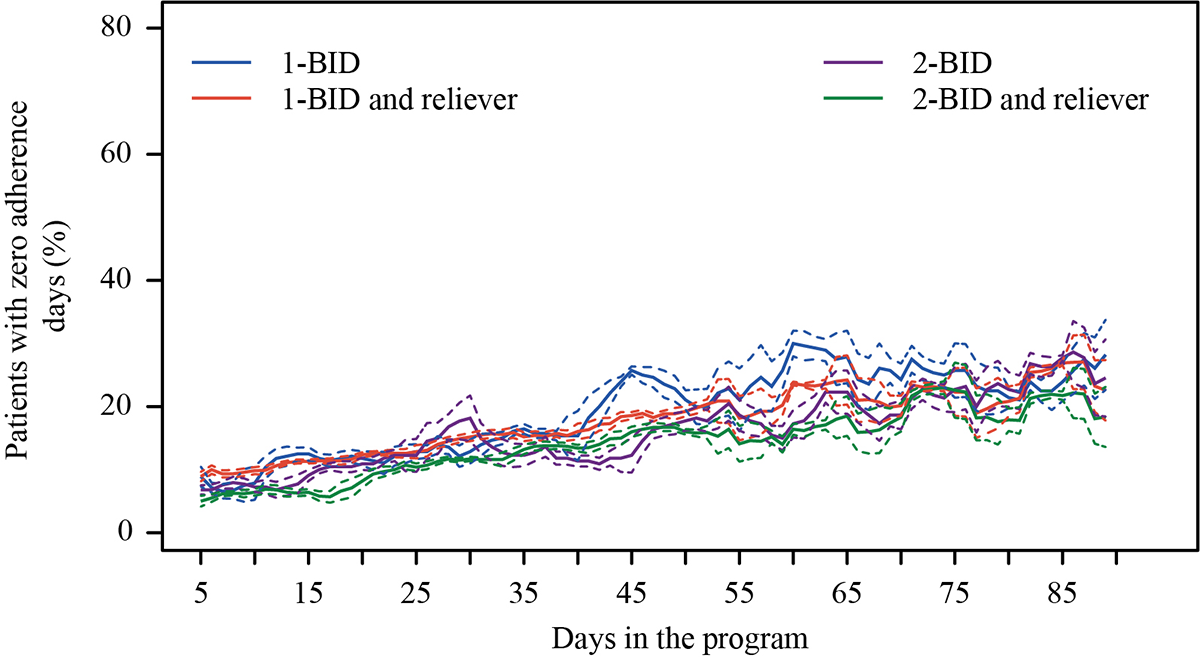

Supplement: Multimedia Appendix 3 [file mhealth_v10i11e25879_app3.png]

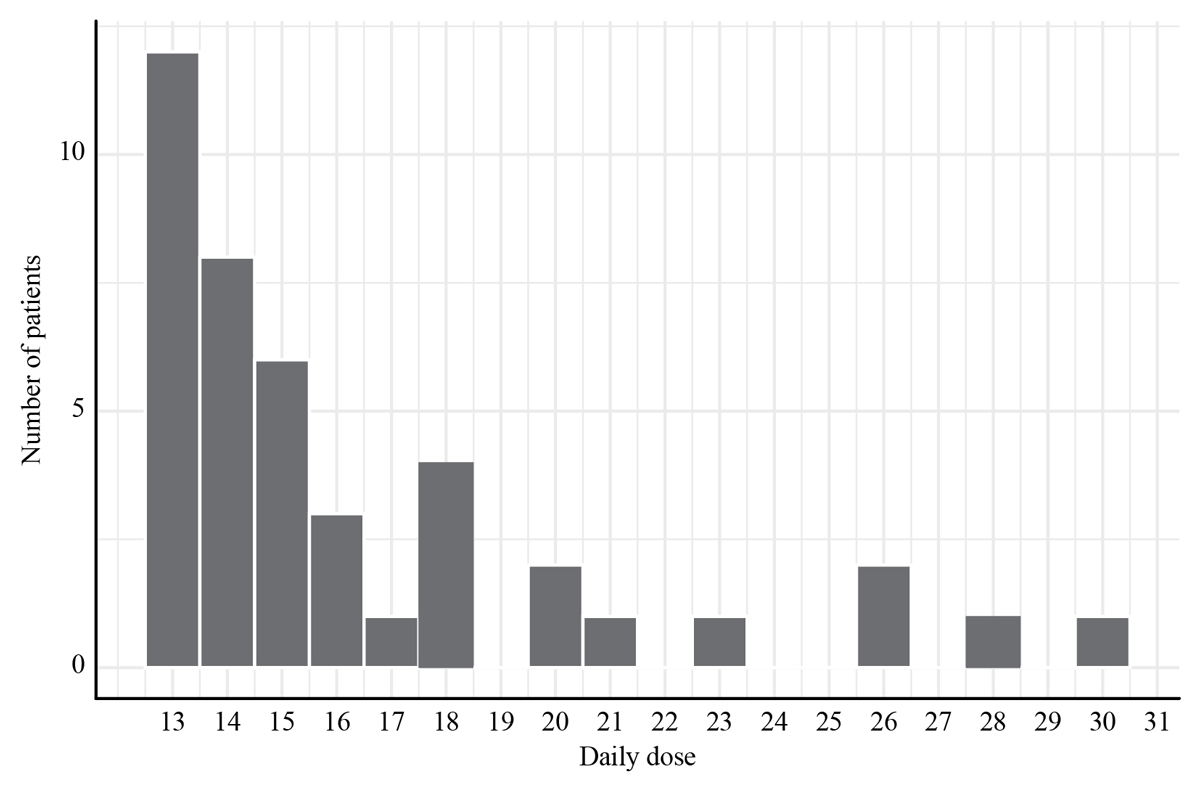

Supplement: Multimedia Appendix 4 [file mhealth_v10i11e25879_app4.png]

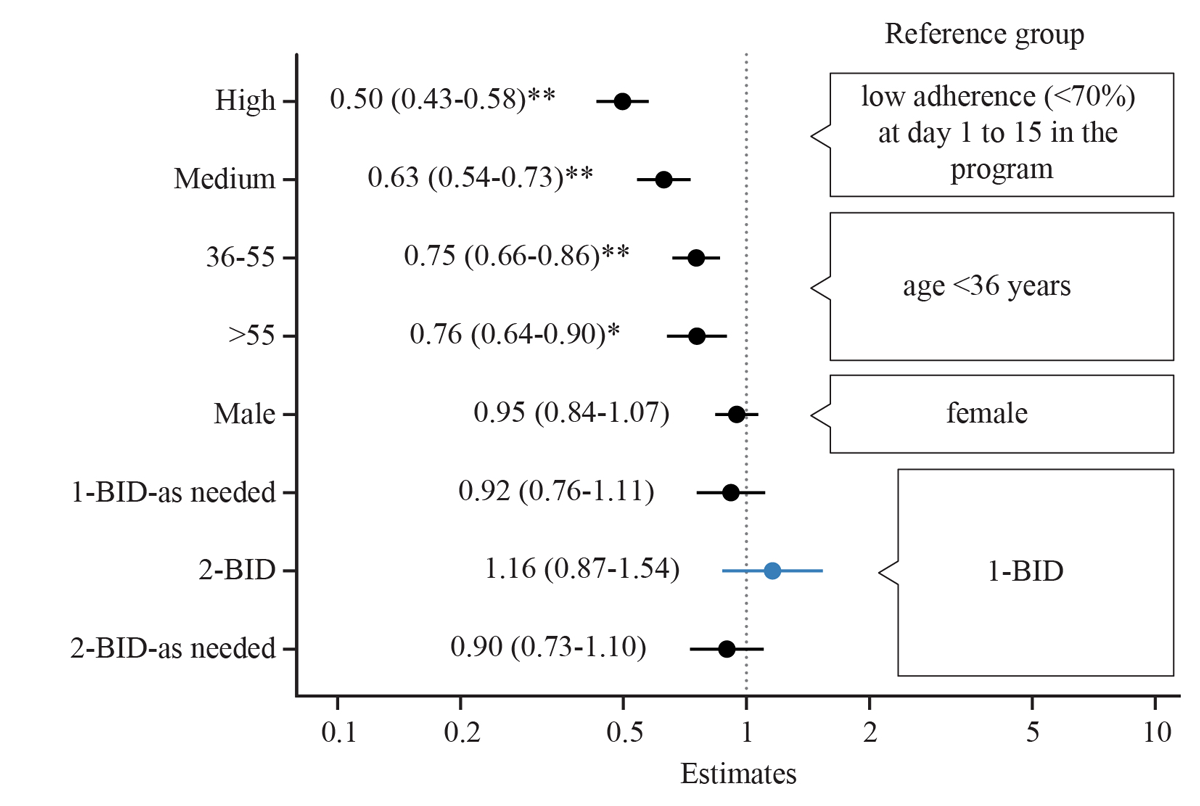

Supplement: Multimedia Appendix 5 [file mhealth_v10i11e25879_app5.png]
