# Supplementary material for: Digital Coaching Using Smart Inhaler Technology to Improve Asthma Management in Patients With Asthma in Italy: Community-Based Study
Source: JMIR Mhealth Uhealth. 2022 Nov 2;10(11):e25879. doi: 10.2196/25879 (PMC9669888; doi:10.2196/25879)
Supplement: Multimedia Appendix 6 [file mhealth_v10i11e25879_app6.docx]

**Multimedia Appendix** **6.** Cox model evaluating factors that contributed the most to discontinuation from the Turbu+ program.

| **Dependent: Surv (disday, status)** | | **All** | **HR^a^ (univariable)** | **HR^a^ (multivariable)** |
| --- | --- | --- | --- | --- |
| **Regimen** | | | | |
|  | 1-BID^b^ | 151 (11.5) | – | – |
|  | 1-BID^b^–as needed | 699 (53.2) | 0.83 (0.69-1.01, *P*=.060) | 0.92 (0.76-1.11, *P*=.37) |
|  | 2-BID^c^ | 91 (6.9) | 1.05 (0.79-1.39, *P*=.729) | 1.16 (0.87-1.54, *P*=.31) |
|  | 2-BID^c^–as needed | 372 (28.3) | 0.81 (0.66-0.99, *P*=.039) | 0.90 (0.73-1.10, *P*=.30) |
| **ADH15^d^** | | | | |
|  | Low | 384 (29.2) | – | – |
|  | Medium | 418 (31.8) | 0.63 (0.54-0.73, *P*<.001) | 0.63 (0.54-0.73, *P*<.001) |
|  | High | 511 (38.9) | 0.49 (0.42-0.56, *P*<.001) | 0.50 (0.43-0.58, *P*<.001) |
| **Age group, years** | |  |  |  |
|  | <36 | 566 (43.1) | – | – |
|  | 36-55 | 502 (38.2) | 0.73 (0.64-0.84, *P*<.001) | 0.75 (0.66-0.86, *P*<.001) |
|  | >55 | 245 (18.7) | 0.72 (0.61-0.85, *P*<.001) | 0.76 (0.64-0.90, *P*=.001) |
| **Sex** | |  |  |  |
|  | Female | 723 (55.1) | – |  |
|  | Male | 590 (44.9) | 0.94 (0.84-1.06, *P*=.331) | 0.95 (0.84-1.07, *P*=.37) |

Cox model excluded those patients who discontinued from the program during the first 15 days; discontinuation is measured from day 16 onwards.

^a^HR: hazard ratio.

^b^1-BID: 1 inhalation twice daily.

^c^2-BID: 2 inhalations twice daily.

^d^ADH15: adherence in 15 days.

Adherence in the first 15 days (proportion of maintenance inhalations taken over this period) was used to categorize patients into low (0%-<70%), medium (70%-<90%), and high (≥90%) adherence groups.
